# Effect of cycloplegia on the measurement of refractive error in Chinese children

**DOI:** 10.1111/cxo.12829

**Published:** 2018-08-22

**Authors:** Tao Li, Xiaodong Zhou, Jie Zhu, Xiaojing Tang, Xiaoyan Gu

**Affiliations:** ^1^ Department of Ophthalmology Jinshan Hospital of Fudan University Shanghai China

**Keywords:** children, Chinese, cycloplegia, refraction

## Abstract

**Background:**

To compare the results of cycloplegic and non‐cycloplegic refractive error measurement in Chinese children, and to assess the relationship between age and the difference in refractive error measured with and without cycloplegia.

**Methods:**

This was a prospective study that recruited 224 healthy Chinese children at an ophthalmology clinic from November 2016 to February 2017. Refraction before and after cycloplegia were measured using an auto‐refractor. Then spherical equivalent M, J_0_, and J_45_ were calculated. The enrolled children were allocated into three groups according to M: myopia, emmetropia, and hyperopia. The distribution of the refraction was further analysed by stratifying by age: four to six years, seven to 11 years, and 12 to 16 years.

**Results:**

Mean non‐cycloplegic M, J_0_, and J_45_ were −1.68 ± 2.00 D, 0.05 ± 0.40 D, and 0.01 ± 0.35 D, while mean cycloplegic M, J_0_, and J_45_ were −1.16 ± 2.17 D, 0.02 ± 0.40 D, and −0.01 ± 0.35 D. Significant differences were found between cycloplegic and non‐cycloplegic M (p = 0.009), whereas there were no significant differences between cycloplegic and non‐cycloplegic J_0_ and J_45_ (p = 0.486 and p = 0.594, respectively). The differences between cycloplegic and non‐cycloplegic M were statistically significant in the four to six years group (p = 0.002) and seven to 11 years group (p = 0.023), whereas there was no significant difference between cycloplegic and non‐cycloplegic M in the 12 to 16 years group (p = 0.151). The proportion of myopia decreased from 78.1 per cent before cycloplegia to 71.4 per cent after cycloplegia, while the proportion of hyperopia increased from 12.1 per cent before cycloplegia to 21.4 per cent after cycloplegia.

**Conclusion:**

Non‐cycloplegic auto‐refraction is found to be inaccurate and not suitable for studies of refractive error in Chinese children.

Myopia is a public health problem, affecting the general population worldwide.^1^, ^2^, ^3^ The common modalities for correcting myopia include spectacles, contact lenses, and surgical procedures. Cycloplegic refraction is considered the gold standard for measuring refractive errors, especially in children.^4^, ^5^, ^6^, ^7^, ^8^ Auto‐refractors are frequently used to obtain an objective refraction in clinical settings, followed by the refinement of vision with subjective refraction, which are also widely used in epidemiological surveys.^9^, ^10^, ^11^

Population‐based studies on the prevalence of refractive error in school‐children have often encountered difficulties in that numerous parents and children refuse to undertake cycloplegic refraction because of the blurred vision and photophobia after cycloplegia. Non‐cycloplegic auto‐refraction has been deemed to overestimate myopia and underestimate hyperopia in children whose accommodative responses are active.^12^, ^13^

Children typically present to an eye clinic because of poor visual acuity. The purpose of this study was to compare the results between cycloplegia and non‐cycloplegia refractive data in Chinese children, and to assess the relationship between age and the differences between both methods.

## Methods

### Subjects

A total of 224 children (224 eyes) were enrolled at the Outpatient Clinic, Department of Ophthalmology, Fudan University Jinshan Hospital, Shanghai, China. The inclusion criteria were: 4–16 years of age; Chinese ethnicity; and normal intraocular pressure (IOP ≤ 21 mmHg). The exclusion criteria were: a history of any surgery or trauma to either eye; amblyopia; strabismus; or systemic diseases.

Refraction data, including sphere (S), cylinder (C), and axis (θ) measurements were analysed by estimating spherical equivalent refraction M, J_0_, and J_45_. These variables were calculated as:

$$M=S+C/2,\;J_{0}=-\left(C/2\right)\;\cos\left(\text{2θ}\right),\;\text{and}$$

$$J_{45}=-\left(C/2\right)\;\sin\left(\text{2θ}\right)$$

with the axis measurement θ expressed in radians.^14^ The enrolled children were allocated into three groups according to M: myopia, emmetropia, and hyperopia. Myopia was defined as M ≤ −0.50 D, emmetropia as −0.50 D < M < +0.50 D, and hyperopia as M ≥ +0.50 D.^15^ The distribution of the refraction was further analysed by stratifying the study children by three age groups: four to six years (preschool children), seven to 11 years (primary school children), and 12 to 16 years (junior middle school children).

All procedures conformed to the tenets of the Declaration of Helsinki. This study was approved by the Ethics Committee of Fudan University Jinshan Hospital, and written informed consent was obtained from the children's parents or guardians.

### Examinations

Comprehensive eye examinations, including uncorrected and corrected distance visual acuity, IOP measurement and auto‐refraction, were performed by trained ophthalmologists and optometrists. Investigators had access to information that could identify individual participants during data collection. IOP was determined by non‐contact tonometry (CT‐80; Canon Inc., Tokyo, Japan).

The principle of auto‐refraction has been demonstrated in detail in previous studies.^7^, ^8^ Briefly, auto‐refraction was performed for each child by the same study optometrist without and with cycloplegia. First, non‐cycloplegic refraction was performed using a Desktop Auto‐refractor (RK‐F1; Canon Inc.) with a measurement range of −20.00 to +20.00 D. Cycloplegia was achieved by topical application of four drops of 0.5 per cent tropicamide (Bausch & Lomb Pharmaceutical Co., Ltd, Shandong, China) at five‐minute intervals.^15^ Auto‐refraction was then performed again using the same equipment by the same optometrist 30 minutes after the last eye drop was administered. Cycloplegia was considered complete if the pupil diameter was more than 6 mm and pupillary constriction was absent. The change of M induced by cycloplegia was calculated as:

Mchange=Mafter cycloplegia–Mbefore cycloplegia.

### Statistical analysis

Statistical analysis was performed using SPSS version 17.0 software (SPSS, Chicago, Illinois, USA). Only data from the right eye of each child were used for analysis. Distributions of refraction data before and after cycloplegia were compared and descriptive statistics including mean, standard error, standard deviation, interquartile range (IQR), skewness, and kurtosis were presented. The distribution of each parameter was assessed using the Kolmogorov–Smirnov test, which revealed that none of the parameters were normally distributed (p < 0.05).

Statistical comparisons in differences between after and before cycloplegic refraction data between groups were made using the Kruskal–Wallis test and post‐hoc Mann–Whitney U‐tests, and its association with age was assessed using Spearman correlation analysis. Chi‐squared tests were used to analyse the difference of proportion of refractive error before and after cycloplegia. All p‐values were two‐sided and considered statistically significant when less than 0.05.

## Results

Of the 224 children, the mean age was 10.1 ± 2.8 years, and 97 (43.3 per cent) were boys. Mean non‐cycloplegic M was −1.68 ± 2.00 D (median: −1.56 D, range: −8.63 D to +8.94 D), and mean cycloplegic M was −1.16 ± 2.17 D (median: −1.12 D, range: −8.50 D to +8.63 D), with a significant difference (p = 0.009) between both values. Mean non‐cycloplegic J_0_ was 0.05 ± 0.40 D (median: 0.00 D, range: −1.47 D to +2.47 D), and mean cycloplegic J_0_ was 0.02 ± 0.40 D (median: 0.00 D, range: −1.47 D to +2.38 D); there was no significant difference (p = 0.486) between both values. Mean non‐cycloplegic J_45_ was 0.01 ± 0.35 D (median: 0.00 D, range: −1.93 D to +1.69 D), and mean cycloplegic J_45_ was −0.01 ± 0.35 D (median: 0.00 D, range: −1.47 D to +1.63 D); there was no significant difference (p = 0.594) between both values. There were no significant differences in M, J_0_ and J_45_ between genders before and after cycloplegia (all p > 0.05).

**Figure 1:**
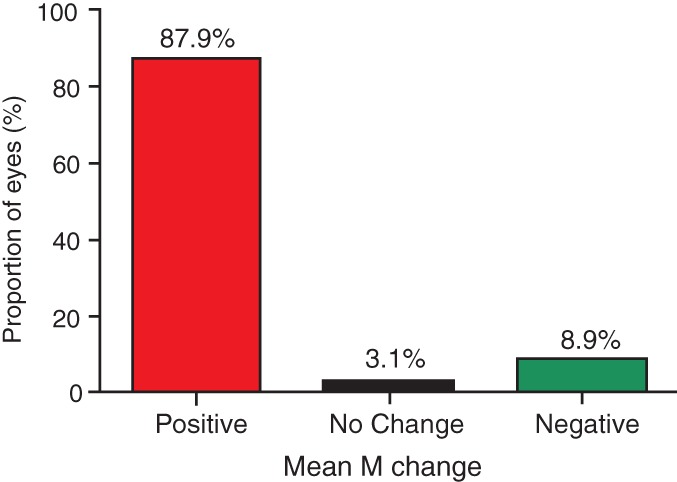
The mean spherical equivalent M change after cycloplegia, compared with non‐cycloplegia. M change = M after cycloplegia – M before cycloplegia.

**Figure 2:**
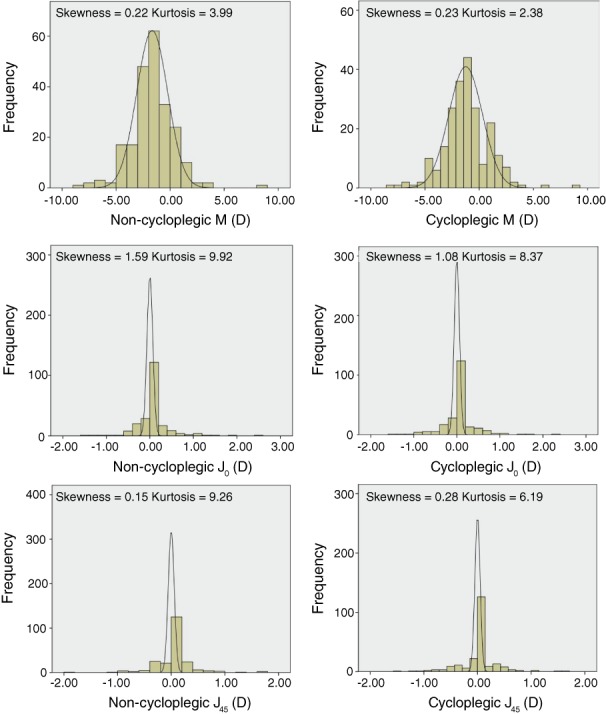
A–B: Distributions of spherical equivalents M, C–D: J_0_ and E–F: J_45_ before and after cycloplegia

Tables 1, 2, 3 show the distributions of M, J_0_ and J_45_ before and after cycloplegia stratified by age and gender. The differences between cycloplegic and non‐cycloplegic M were statistically significant in the four to six years group (p = 0.002) and seven to 11 years group (p = 0.023), whereas there was no significant difference between cycloplegic and non‐cycloplegic M in the 12 to 16 years group (p = 0.151). Furthermore, the differences between cycloplegic and non‐cycloplegic J_0_ and J_45_ were not statistically significant (all p *>* 0.05). Figure 1 shows the change in mean M before and after cycloplegia. In 197 eyes (87.9 per cent), cycloplegia led to a positive shift in M values, whereas a negative shift in M values occurred in 20 eyes (8.9 per cent). The distribution of M, J_0_ and J_45_ were more negatively skewed before cycloplegia (Figure 2).

**Table 1:** Distributions of spherical equivalent M before and after cycloplegia, stratified by age and gender

	Cycloplegia	Mean (D)	Standard error	Standard deviation (D)	Skewness	Kurtosis	IQR (D)
Overall							
	Before	−1.68	0.13	2.00	0.22	3.99	2.06
	After	−1.16	0.14	2.17	0.23	2.38	2.10
Age (years)							
4–6	Before	0.10	0.17	0.89	−0.34	−0.97	1.68
	After	0.97	0.21	1.11	−0.49	−0.45	1.31
7–11	Before	−1.52	0.18	1.98	1.11	6.09	1.90
	After	−0.97	0.19	2.10	1.04	3.62	2.00
12–16	Before	−2.65	0.21	1.79	−1.14	1.59	2.19
	After	−2.31	0.22	1.86	−1.13	1.61	2.26
Gender							
Boys	Before	−1.69	0.18	1.79	−0.45	1.27	2.18
	After	−1.29	0.20	1.99	−0.20	0.76	2.53
Girls	Before	−1.68	0.19	2.16	0.51	4.78	2.00
	After	−1.07	0.20	2.30	0.41	2.96	1.88

D: dioptres, IQR: interquartile range.

**Table 2:** Distributions of J_0_ before and after cycloplegia, stratified by age and gender

	Cycloplegia	Mean (D)	Standard error	Standard deviation (D)	Skewness	Kurtosis	IQR (D)
Overall							
	Before	0.05	0.03	0.40	1.59	9.92	0.16
	After	0.02	0.03	0.40	1.08	8.37	0.06
Age (years)							
4–6	Before	0.03	0.13	0.70	0.43	1.82	0.59
	After	−0.05	0.12	0.63	0.48	1.19	0.47
7–11	Before	0.06	0.03	0.37	2.83	15.58	0.10
	After	0.01	0.04	0.40	1.60	11.67	0.08
12–16	Before	0.04	0.03	0.28	0.74	3.56	0.14
	After	0.07	0.03	0.25	1.43	2.84	0.07
Gender							
Boys	Before	0.08	0.04	0.38	1.57	5.94	0.22
	After	0.02	0.04	0.39	−0.23	3.47	0.08
Girls	Before	0.03	0.04	0.41	1.65	12.69	0.11
	After	0.02	0.04	0.41	1.97	11.76	0.06

D: dioptres, IQR: interquartile range.

**Table 3:** Distributions of J_45_ before and after cycloplegia, stratified by age and gender

	Cycloplegia	Mean (D)	Standard error	Standard deviation (D)	Skewness	Kurtosis	IQR (D)
Overall							
	Before	0.01	0.02	0.35	1.15	9.26	0.10
	After	−0.01	0.02	0.35	0.28	6.19	0.04
Age (years)							
4–6	Before	0.06	0.11	0.59	1.32	2.95	0.44
	After	0.06	0.13	0.66	0.23	1.97	0.47
7–11	Before	0.01	0.03	0.36	−1.11	8.33	0.11
	After	−0.02	0.03	0.31	−0.27	3.37	0.01
12–16	Before	−0.02	0.02	0.17	−0.58	1.44	0.56
	After	−0.01	0.03	0.22	0.53	1.41	0.11
Gender							
Boys	Before	0.04	0.04	0.40	0.36	10.87	0.17
	After	−0.02	0.04	0.39	0.51	7.36	0.10
Girls	Before	−0.02	0.03	0.30	−0.43	3.33	0.08
	After	0.00	0.03	0.32	−0.02	3.80	0.00

D: dioptres, IQR: interquartile range.

**Figure 3:**
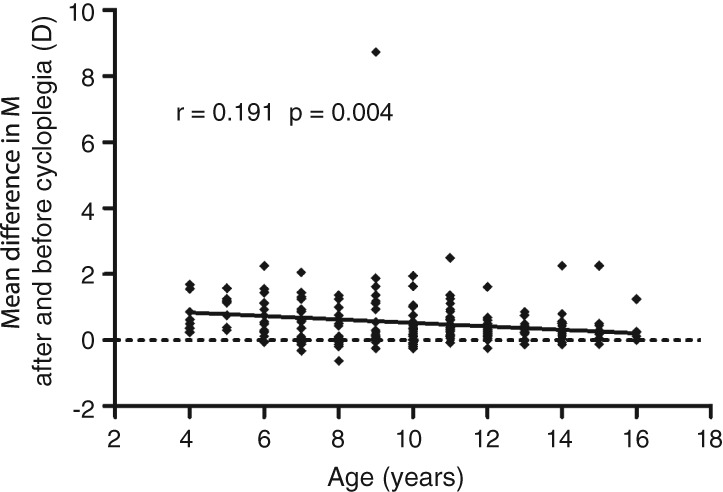
Mean difference in M after and before cycloplegia correlated negatively (but weakly) with age (r = 0.191, p = 0.004)

**Figure 4:**
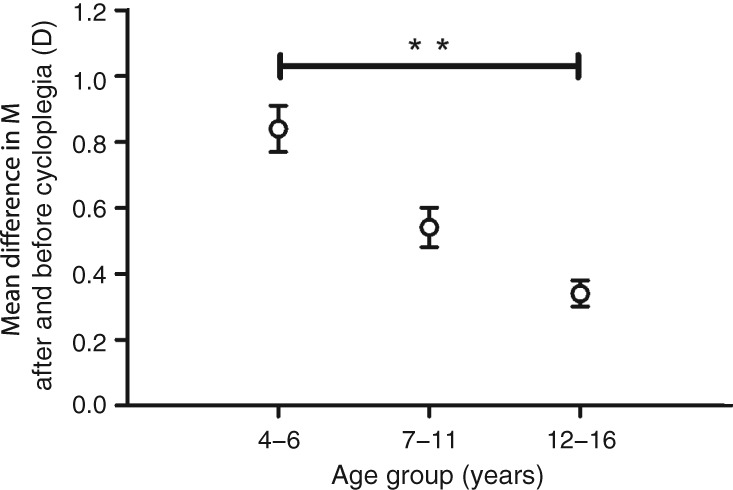
Distribution of mean difference between after and before cycloplegia, stratified by age. **p < 0.01.

The mean difference in M after and before cycloplegia was 0.52 ± 0.77 D (median: 0.31 D, range: −0.63 D to +8.74 D). Figure 3 shows that the mean difference between cycloplegic and non‐cycloplegic refraction correlated negatively (but weakly) with age (r = 0.191, p = 0.004). The mean M differences varied significantly among different age groups (Figure 4, p = 0.009). Post‐hoc analyses showed that the mean M differences were significantly greater in four to six years group than those in 12 to 16 years group (p = 0.007), whereas there were no significant differences in M between the seven to 11 years group and the other two groups (both p > 0.05).

Table 4 compares the proportions of children with myopia, emmetropia and hyperopia based on non‐cycloplegic and cycloplegic M. The proportions are shown in the overall study children and then stratified by age. Comparing the proportion of myopia in the non‐cycloplegic condition and the cycloplegic condition revealed that only 91.4 per cent of all eyes with a non‐cycloplegic myopic refractive error remained myopic under cycloplegic refractometry, whereas the remaining 8.6 per cent of eyes became emmetropic or hyperopic under cycloplegia.

**Table 4:** Proportion of spherical equivalent M before and after cycloplegia

Age (years)		Proportion, n (%), before cycloplegia	Proportion, n (%), after cycloplegia	p
Overall	Myopia	175 (78.1)	160 (71.4)	0.024
	Emmetropia	22 (9.8)	16 (7.1)	
	Hyperopia	27 (12.1)	48 (21.4)	
4–6	Myopia	8 (29.6)	5 (18.5)	0.032
	Emmetropia	8 (29.6)	2 (7.4)	
	Hyperopia	11 (40.7)	20 (74.1)	
7–11	Myopia	99 (78.6)	90 (71.4)	0.240
	Emmetropia	11 (8.7)	10 (7.9)	
	Hyperopia	16 (12.7)	26 (20.6)	
12–16	Myopia	68 (95.8)	65 (91.5)	0.331
	Emmetropia	3 (4.2)	4 (5.6)	
	Hyperopia	0 (0.0)	2 (2.8)	

The proportion of myopia decreased from 78.1 per cent before cycloplegia to 71.4 per cent after cycloplegia, and the proportion of emmetropia decreased from 9.8 per cent before cycloplegia to 7.1 per cent after cycloplegia. Furthermore, the proportion of hyperopia increased from 12.1 per cent before cycloplegia to 21.4 per cent after cycloplegia. The proportion of myopia was 29.6 per cent in the four to six years group, 78.6 per cent in the seven to 11 years group and 95.8 per cent in the 12 to 16 years group, which decreased to 18.5 per cent in the four to six years group, 71.4 per cent in the seven to 11 years group and 91.5 per cent in the 12 to 16 years group after cycloplegia. Furthermore, the proportion of hyperopia was 40.7 per cent in the four to six years group, 12.7 per cent in the seven to 11 years group and 0.0 per cent in the 12 to 16 years group, which increased to 74.1 per cent in the four to six years group, 20.6 per cent in the seven to 11 years group and 2.8 per cent in the 12 to 16 years group after cycloplegia.

## Discussion

In the present study, cycloplegia resulted in less myopic/more hyperopic mean M, whereas cycloplegia did not have a significant effect on J_0_ and J_45_. In addition, there was a greater pre‐ and post‐cycloplegic difference in M values in four to six year old children compared with older children. Due to a significant misclassification of myopia and hyperopia without cycloplegia, the necessity of cycloplegia should be taken seriously during refraction examination in children.

The results of our study agree with previous investigations in that non‐cycloplegic auto‐refraction appears to be too unreliable to assess the refractive error in children.^5^, ^6^, ^16^ In the present study, cycloplegia led to less myopia or greater hyperopia with auto‐refraction, with a mean difference of 0.52 D in M among children aged four to 16 years. Hu et al.,^7^ Zhu et al.^8^ and Zhao et al.^17^ reported a mean difference of 0.78 D, 0.57 D and 1.23 D after and before cycloplegia among Chinese school‐aged children, respectively.

A comparison in a wide age range between cycloplegic and non‐cycloplegic refraction data showed that the difference in mean M with and without cycloplegia fell from 0.71 D in those aged five to 10 years to 0.14 D in those over 70 in an Iranian population.^18^ However, no significant difference between cycloplegic and non‐cycloplegic J_0_ and J_45_ data were found in the present study, which was in agreement with the finding reported by Zhao et al.^17^

There was little difference between noncycloplegic and cycloplegic measurements of astigmatism: mean values for J_0_ and J_45_ differences were −0.08 ± 0.13 D and −0.01 ± 0.09 D, respectively.^17^ These inter‐study disparities may be due to different characteristics of the study participants (such as age and ethnicity) and methodological issues (such as cycloplegia methods). For example, cyclopentolate was used for cycloplegia in the previous Chinese studies,^7^, ^8^, ^17^ while tropicamide was used in the present study. Lovasik et al.^19^ reported that the cycloplegic effect of tropicamide was much less than that of cyclopentolate in children, while Hofmeister et al.^20^ found that tropicamide may be equally efficacious for refractive measurements with cyclopentolate. Furthermore, there were no significant gender differences in non‐cycloplegic and cycloplegic refraction data.

The mean M difference between cycloplegic and non‐cycloplegic refraction correlated negatively (but weakly) with age with auto‐refraction in the present study. This finding is similar to that reported by Fotouhi et al.,^18^ which indicated that age played an important role in accommodation and the M changed after cycloplegia. The influence of accommodation on refraction increases with younger age.

Accommodation may partially or fully correct for existing hyperopia. Furthermore, younger children with hyperopia generally have greater accommodative efforts in comparison to myopic children with relatively lower accommodation requirements.^7^ Older children were more likely to be myopic in the present study, with correspondingly smaller differences between non‐cycloplegic and cycloplegic measurements.

Although cycloplegic refraction produced more hyperopic results as well as fewer myopes in the present study, a small proportion (20 eyes, 8.9 per cent) of the children became even more myopic or less hyperopic after cycloplegia. The foremost reasons for this phenomenon can be analysed as follows. First, cycloplegia might be inadequate and residual accommodation after cycloplegia might exist when refraction of these study children were measured by auto‐refractors. Although the reliability of measuring refractive error using auto‐refractors had been tested in previous studies,^9^, ^21^, ^22^, ^23^, ^24^ the present authors endeavoured to minimise this measurement error by allowing enough time for cycloplegia, and by measuring refractive error using the same equipment and by the same optometrist in this study.

In addition, spherical aberration might be responsible for the negative change of M. Spherical aberration significantly increased after cycloplegia,^25^, ^26^ which was negatively correlated with axial myopia.^27^ Gao et al.^28^ reported that cycloplegia induced significant decreases in the lens thickness and backward movement of the lens, which increased the spherical aberration.

Cycloplegia could relax the ciliary muscle tonus and eliminate continuous accommodation. Due to the influence of accommodation before cycloplegia, studies with non‐cycloplegic refractometry overestimated the prevalence of myopia and underestimated hyperopia.^21^, ^22^ The results of the present study demonstrated that not all the eyes with non‐cycloplegic myopic refractive error remained myopic under cycloplegia, whereas some eyes became emmetropic or hyperopic.

In different age groups, cycloplegia decreased the proportion of myopia and increased the proportion of hyperopia. Furthermore, the proportion of myopia in the present study was higher than that reported by a previous population‐based study.^29^ This could be mainly explained by the fact that children from the eye clinic in the present study were more likely to have visual acuity problems (such as myopia), compared with the participants of population‐based studies. Children with good uncorrected distance visual acuity would seldom go and have their eyes checked at ophthalmology clinics.

There may be two limitations in the present study. Four drops of 0.5 per cent tropicamide were used to achieve cycloplegia in Chinese children with dark‐brown irides. Since the effect of cycloplegic drugs depend on iris colour, which is mainly determined by genetic ancestries, our findings may not be directly generalised to other populations. In addition, measurement errors may have occurred using auto‐refraction as previously mentioned, which may have misrepresented the results. Harvey et al.^30^ reported that non‐cycloplegic auto‐refraction provided reliable measurements of refractive error in young children, but there was large variability in validity.

In summary, non‐cycloplegic auto‐refraction is found to be inaccurate and not suitable for studies of refractive error in Chinese children. Lack of cycloplegia is associated with significant misclassifications in both myopia and hyperopia. Age plays an important role in the refraction change after cycloplegia. Accurate refraction must be measured after cycloplegia in young children.
